# PlasmoDraft: a database of *Plasmodium falciparum *gene function predictions based on postgenomic data

**DOI:** 10.1186/1471-2105-9-440

**Published:** 2008-10-16

**Authors:** Laurent Bréhélin, Jean-François Dufayard, Olivier Gascuel

**Affiliations:** 1 Projet Méthodes et Algorithmes pour la Bioinformatique, LIRMM, Univ. Montpellier 2, CNRS, 161 rue Ada, 34392 MONTPELLIER, France

## Abstract

**Background:**

Of the 5 484 predicted proteins of *Plasmodium falciparum*, the main causative agent of malaria, about 60% do not have sufficient sequence similarity with proteins in other organisms to warrant provision of functional assignments. Non-homology methods are thus needed to obtain functional clues for these uncharacterized genes.

**Results:**

We present PlasmoDraft , a database of Gene Ontology (GO) annotation predictions for *P. falciparum *genes based on postgenomic data. Predictions of PlasmoDraft are achieved with a *Guilt By Association *method named Gonna. This involves (1) a predictor that proposes GO annotations for a gene based on the similarity of its profile (measured with transcriptome, proteome or interactome data) with genes already annotated by GeneDB; (2) a procedure that estimates the confidence of the predictions achieved with each data source; (3) a procedure that combines all data sources to provide a global summary and confidence estimate of the predictions. Gonna has been applied to all *P. falciparum *genes using most publicly available transcriptome, proteome and interactome data sources. Gonna provides predictions for numerous genes without any annotations. For example, 2 434 genes without any annotations in the Biological Process ontology are associated with specific GO terms (*e.g*. Rosetting, Antigenic variation), and among these, 841 have confidence values above 50%. In the Cellular Component and Molecular Function ontologies, 1 905 and 1 540 uncharacterized genes are associated with specific GO terms, respectively (740 and 329 with confidence value above 50%).

**Conclusion:**

All predictions along with their confidence values have been compiled in PlasmoDraft, which thus provides an extensive database of GO annotation predictions that can be achieved with these data sources. The database can be accessed in different ways. A global view allows for a quick inspection of the GO terms that are predicted with high confidence, depending on the various data sources. A gene view and a GO term view allow for the search of potential GO terms attached to a given gene, and genes that potentially belong to a given GO term.

## Background

Malaria is one of the most prevalent disease in the world, infecting 400 million people every year, and causing 2.7 million deaths, mainly children under 5 years [1]. *Plasmodium falciparum*, the main causative agent of this parasitic disease, develops drug resistance and no effective vaccine is available. Of the 5 484 coding genes of *P. falciparum *( version 5.4), about 60% do not have sufficient similarity to proteins in other organisms to warrant provision of functional assignments. Thus, almost two-thirds of the proteins appear to be specific to *P. falciparum*, a much higher proportion than observed in other enkaryotes [2]. However, this is likely exacerbated by the high evolutionary distance between *P. falciparum *and other sequenced eukaryotes, so homology detection is a hard task. Because of the extreme AT bias (80%), the high amino acid bias (six amino acids account for more than 50% of the protein composition) and the presence of a large number of low complexity repeat regions that are believed to form non-globular segments [3], standard sequence comparison methods based on BLAST [4] or HMMER [5] could be ineffective [6]. Non-homology methods are thus needed to obtain functional clues for these uncharacterized genes [7].

Methods based on post-genomic data (mainly gene expression and protein interaction) have been proposed. These are commonly called *Guilt by Association *(GBA) methods. Contrary to sequence homology which involves inter-species annotation transfers, *i.e*. genes characterized in other species are used to annotate genes of the newly sequenced genome, GBA approaches involve intra-species annotation transfers: the genes already characterized in the genome, *e.g*. by wet experiments or using sequence homology, are used for the annotation of the other genes (guilt by association principle). Gene expression data are often used, since genes with similar transcriptomic profiles likely share common functional roles [8,9]. In the same way, protein interaction data are also used since proteins that share common interactors likely share common functions [10-12]. These methods provide functional predictions for the uncharacterized genes, and new clues to be compared with the predictions achieved by homology.

Part of these new post-genomic methods work in a non-supervised way: first a gene clustering algorithm is run on the post-genomic data to cluster the genes into several groups. Then, in each cluster and for each potential function, a statistical test is applied to compare the proportion of genes annotated with this function in the cluster with that in the complete set of genes. Functions that appear to be over-represented in one cluster are used to annotate the uncharacterized genes that belong to this cluster. Several genome-scale studies have been conducted using this principle, *e.g*. [8,13,14].

Some other GBA methods work in a supervised way: first, based on the post-genomic data of already characterized genes, a supervised learning algorithm is run to learn a predictor, *i.e*. a function that takes post-genomic measurements of a given gene as input, and outputs one or several functional predictions for that gene. This predictor is then used to annotate the uncharacterized genes. Typical examples of this approach are, *e.g*. [11,15,16]. Zhou et al. [17] presented OPI, a supervised method that predicts Gene Ontology annotations using gene expression profiles and was applied on *P. falciparum*. Alternative methods work in a semi-supervised way [18]; these use gene clustering as in the non-supervised approach, but clustering is not fully unsupervised as the function of the already characterized genes is used to define the clusters.

In this paper, we present PlasmoDraft , a database of Gene Ontology (GO) annotation predictions for *P. falciparum *achieved by applying a GBA predictor named Gonna (for *Gene Ontology Nearest Neighbor Approach*) on several transcriptome (microarray), proteome (mass-spectrometry) and protein-protein interaction data. The Gonna system involves: (1) a supervised k-nearest-neighbor predictor that proposes predictions on the basis of each data source; (2) a cross-validation procedure that estimates the confidence of the predictions achieved with each data source; (3) a procedure that combines the results achieved with the different data sources to estimate a global confidence value of each prediction for each gene. The PlasmoDraft database provides all of these predictions along with their confidence values in a friendly interface that allows easy browsing and querying.

## Methods

Gonna proposes annotation predictions in the GO framework. The GO Consortium  has developed a systematic and standardized nomenclature to annotate genes in terms of their associated biological processes (BP), cellular components (CC) and molecular functions (MF), in a species-independent manner. Each ontology describes generalization relationships between hundreds of terms. The most general term is at the top of the ontology, while the bottom terms are the most specific ones. A gene may be annotated with several GO terms of the same ontology. Moreover, due to the generalization relationship, when a gene is annotated with a term *t*, then it is also annotated with all upper terms that generalize *t *(a principle known as the "*true path rule*" in GO context). In PlasmoDraft, the specificity of a term is evaluated by its prior probability, *i.e*. the proportion of already characterized genes of *P. falciparum *that belong to this term. In this way, the leaves of the ontologies are the most specific terms with low prior probabilities, while the root of the ontology is the most common term with a prior probability of 1. Gonna uses the GO annotations available on PlasmoDB and provided by GeneDB as prior knowledge database to propose new annotations. The GO consortium distinguishes between curator-assigned annotations and automatically-assigned annotations. Curator-assigned annotations involve annotations that come from experimental data (GO evidence codes IDA, IPI, etc.), or that have been inferred by sequence similarity and curated by an expert (GO evidence code ISS). Automatically-assigned annotations involve all electronically inferred annotations (usually by sequence similarity) that have not been reviewed by an expert (GO evidence code IEA). Here, due to the scarcity of the curator-assigned annotations for *P. falciparum *(~60% annotations possess IEA evidence code only), all available GO annotations are considered, without regard to their evidence code (this choice is further-discussed below). Every gene with an annotation in the considered ontology (whatever its evidence code) is then referred as "characterized".

### The predictor

Gonna uses a *k*-nearest neighbor approach [19]. It takes as input two positive integers *K *and *K' *≤ *K *(*e.g. K *= 6 and *K' *= 4), one ontology (MF, BP, or CC), and one postgenomic data source *D *(*e.g*. the microarray data of [14]). With this data source, Gonna computes a function *S*_*D *_that measures the similarity *S*_*D *_(*g*, *h*) of every gene pair (*g*, *h*). For example, if *D *is a transcriptomic data set then *S*_*D *_measures the similarity of profiles using the Pearson correlation coefficient. When asked for the GO categories of a gene *g*, Gonna uses the *S*_*D *_function to search for the *K *genes already characterized in the selected ontology by GeneDB, which have the highest level of similarity with *g*. Then, for each GO term *t *of the ontology, Gonna looks at these *K *genes, and if at least *K' *are associated with *t*, then *g *is predicted to be also associated with *t*; otherwise *g *is not considered to be in *t*. Note that when looking at the terms associated with the neighbor genes, Gonna considers all the upper terms generalizing the direct annotations (*i.e*. all terms in the true path rule).

Some choices are critical to insure that Gonna provides relevant and accurate predictions. The first critical choice is related to the similarity measure, which has to capture the "signature" of the gene functions in the data set at hand. When two genes appear to be similar, this should imply that they share common functions. For transcriptomic (microarray) and proteomic (mass-spectrometry) data, we use the Pearson correlation coefficient that gives high similarity to genes with correlated transcriptomic/proteomic profiles. Other similarity measures, as the classical Euclidean metric, could be possible, but the Pearson correlation measure has been shown to perform well to detect functional links in several analyses [20]. For the protein-protein interaction data, we use the Czekanovski-Dice metric [21], which gives high similarity to pairs of genes that share many interactors, and has been shown to perform well to predict biological functions [10].

Another critical choice is related to the *K *and *K' *values. *K *should be neither too large (else some neighbors will not be similar to the studied gene) nor too low (to avoid reduced, non-representative gene samples). With *K' *the problem is different. If *K' *is high (close to *K*), then the proportion of good predictions is likely to be high, but only a few predictions could be achieved on the most specific terms of the ontology, and most of the predictions would involve the most general (and hence less interesting) terms. Conversely, if *K' *is low, then the proportion of good predictions declines, but more predictions are made on the most specific terms. In PlasmoDraft, we use two pairs of parameters (*K*, *K'*) for each postgenomic data source: one stringent pair (*K *= 6, *K' *= 4) is used to achieve, for each GO term, a first set of predictions that usually has a high proportion of good predictions (see next section for an estimate of this proportion). Next, a second, non-stringent pair (*K *= 6, *K' *= 2) is used to come up with, for each GO term, another set of predictions that cannot be achieved with the stringent setting, but which usually contains a lower proportion of good predictions.

**Figure 1:**
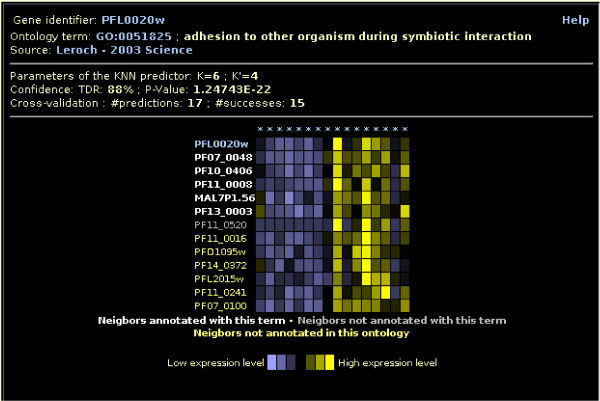
**The neighbors view**. Profiles of the *K *nearest characterized neighbors that support (white), or does not support (gray), the prediction of gene PFL0020w in term Adhesion to other organism during symbiotic interaction (GO:0051825) for the Leroch et al. (2003) data source [14]. For comparison purpose, profiles of the *K *nearest uncharacterized neighbors (yellow) are also reported.

This *k*-nearest neighbor predictor has several appealing features. It is a direct and simple implementation of the GBA principle, which allows the predictions to be explained by exhibiting the *K' *genes annotated by GeneDB that support each prediction (see Figure 1). In fact, Gonna uses a basic principle similar to gene expression mining tools as g:profiler [22], which help users to make their own predictions. These tools search for genes with expression profile correlated with that of the studied gene, look for GO terms enriched in the neighboring gene list, and then predict the selected GO terms for the studied gene. Gonna can thus be viewed as a systematic and automatic implementation of this natural principle, combined with confidence estimation and data source aggregation (see below). Moreover, Gonna can be used with any present and future postgenomic data source, as long as there is a relevant similarity measure. Next. Gonna is consistent with the structure of the ontology. This important property means that if any gene is predicted in a GO term *t*, then it must be predicted in all terms that generalize *t*. Finally, Gonna has low computing time, which enables intensive use of the cross-validation procedure to assess the confidence of the predictions.

### Assessing the predictions

Cross-validation (CV) is a well known procedure to estimate the error rate of supervised classification methods [19]. The leave-one-out version of CV, which we use here, involves: (1) running Gonna on each gene already characterized in GeneDB as if it were an uncharacterized gene, and (2) comparing the predictions to the true annotations. Since no functional information on this gene is supplied to Gonna for the predictions, this procedure provides an unbiased estimate of the method performance [19]. For a given GO term *t*, the correct predictions in CV involve the genes predicted in *t*, which are already annotated by this term in GeneDB (or by a specialization of *t*, cf. the true path rule); the wrong predictions involve the genes predicted in *t *which are already annotated in the ontology under consideration (MF, BP, or CC) but not with *t *(or one of its specializations). Genes without any annotation in the selected ontology are not taken into account. It is convenient to present all of these quantities in tabular form (see Table 1). For example, *p*_*a *_denotes the number of genes predicted in the GO term *t *which are annotated with *t *in GeneDB, while *n*_*n *_denotes the number of genes not annotated with *t *which have not been predicted in *t *in CV. Then, the True Discovery Rate (*TDR*) associated with this GO term (and for a given data source) is estimated by

(1) *TDR *= *p*_*a*_/(*p*_*a *_+ *p*_*n*_).

**Figure 2:**
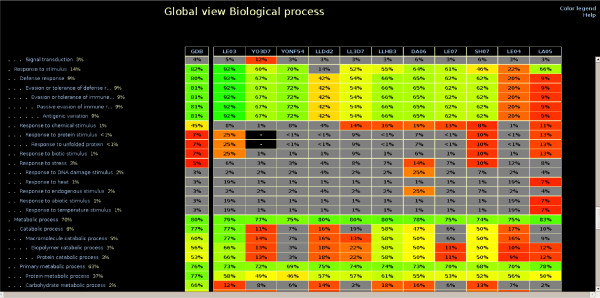
**An extract of the Biological Process global view**. This view presents a summary of all of the best *GDB*s and *TDR*s that are associated with each GO term and data source. Clicking on any term opens the corresponding GO term view.

For example, a GO term with a *TDR *of 80% means that when Gonna predicts that a gene belongs to this term, this prediction has 80% chance of being correct. Note that due to the incompleteness of the annotations, the above formula may be a conservative estimate of the *TDR*, because some predictions considered as wrong may actually be correct. Moreover, when the sample size (*p*_*a *_+ *p*_*n*_) is low. this *TDR *estimate may not be fully accurate. So, we also compute the p-value of achieving by chance *p*_*a *_or more correct predictions (among *p*_*a *_+ *p*_*n*_) if the true *TDR *were equal to the prior probability of the term. If this p-value is higher than 5%, then the *TDR *is not considered to be significantly higher than the prior probability. PlasmoDraft reports the *TDR*s of the predictions with a color code that ranges from red (0%) to light green (100%) via yellow (50%), while non-significant *TDR*s appear in gray (cf Figure 2). As described above, two sets of predictions are achieved for each GO term and data source using two parameters *K'*. Therefore, one *TDR *is estimated for each of these sets: the first *TDR *reports the accuracy of the predictions achieved with the stringent predictor, while the second *TDR *reports the accuracy of the predictions achieved with the non-stringent predictor but which are not supported by the stringent one. As expected, the first *TDR *is usually higher than the second one. When neither the stringent predictor nor the non-stringent one apply, the gene is said to be "non predicted in *t*".

**Table 1:** Correct and wrong predictions associated with a given GO term *t *in the CV procedure

	predicted in	not predicted in
annotated with	*p*_*a*_	*n*_*a*_
not annotated with	*p*_*n*_	*n*_*n*_

*p*_*a *_and *p*_*n *_denote the number of genes predicted in the GO term *t *which are, and are not, annotated with *t *in GeneDB, respectively. *n*_*a *_and *n*_*n *_denote the number of genes not predicted in *t *which are. and are not, annotated with *t*, respectively.

The advantage of estimating the *TDR *of each GO term rather than estimating a global performance on the whole ontology is that it allows to differentiate GO terms that appear well suited for applying a GBA approach with the considered data source. Indeed, all GO terms cannot be predicted with the same accuracy. First because some terms are more general than others (and thus are *a priori *more likely). But also because some functions (GO terms) have a more apparent signature than others in the considered data source. For example, while they have a similar prior probability (~10%), GO terms antigenic variation (GO:0020033) and post-translational protein modification (GO:0043687) get 90% and 15% *TDR*s with the microarray data of [14], respectively.

### Combining the data sources

When each data source has been used to produce predictions, and *TDR*s have been estimated for each GO term and each source, Gonna combines all of these results to propose a Global Degree of Belief (*GDB*) for each prediction. If gene *g *has been predicted to be associated with GO term *t *by one or several sources, Gonna computes the *GDB *of this prediction in the following way. Let 1,..., *n *and *n *+ 1,..., *m *denote the data sources that support, and do no support, the prediction of *g *in *t*, respectively. We use the notation *d*_*i *_and *¬d*_*j *_to indicate that data sources *i *and *j *support and do not support the prediction of *g *in *t*, respectively. We first compute a global confidence score that is a rough estimate of the probability that the prediction is correct, given that it is supported by data sources 1,..., *n *but not by data sources *n *+ 1,..., *m*. Using Bayes theorem, this probability can be written as

$$\begin{array}{l} P(t|d_{1},...,d_{n},\neg d_{n+1},...,\neg d_{m})=\\ \frac{P(d_{1},...,d_{n},\neg d_{n+1},...,\neg d_{m}|t)\times P(t)}{P(d_{1},...,d_{n},\neg d_{n+1},...,\neg d_{m})}. \end{array}$$

*P*(*t*) is the prior probability of term *t *(estimated by the proportion of already characterized genes of *P. falciparum *that belong to *t*). *P*(*d*_1_,..., *d*_*n*_, ¬*d*_*n*+1_,..., ¬*d*_*m*_|*t*) is the probability that data sources 1,..., *n *and data sources *n *+ 1,..., *m *support and do not support the prediction of *g *in *t *when *g *belongs to *t*, respectively. We use the conditional independence assumption [19] to estimate this latter term and the probability *P*(*d*_1_,..., *d*_*n*_, ¬*d*_*n*+1_,..., ¬*d*_*m*_):

$$\begin{array}{l} P(d_{1},...,d_{n},\neg d_{n+1},...,\neg d_{m}|t)≃\\ P(d_{1}|t)\times \cdots \times P(d_{n}|t)\times \\ \;P(\neg d_{n+1}|t)\times \cdots \times P(\neg d_{m}|t), \end{array}$$

$$\begin{array}{l} P(d_{1},...,d_{n},\neg d_{n+1},...,\neg d_{m}|\neg t)≃\\ P(d_{1}|\neg t)\times \cdots \times P(d_{n}|\neg t)\times \\ \;P(\neg d_{n+1}|\neg t)\times \cdots \times P(\neg d_{m}|\neg t), \end{array}$$

and

$$\begin{array}{l} P(d_{1},...,d_{n},\neg d_{n+1},...,\neg d_{m})=\\ P(d_{1},...,d_{n},\neg d_{n+1},...,\neg d_{m}|t)P(t)+\\ \;P(d_{1},...,d_{n},\neg d_{n+1},...,\neg d_{m}|\neg t)P(\neg t). \end{array}$$

Terms *P*(*d*_*i*_|*t*), *P*(¬*d*_*i*_|*t*), *P*(*d*_*i*_|¬*t*), and *P*(¬*d*_*i*_|¬*t*) are estimated with the quantities computed in the CV and displayed in Table 1. For example, *P*(*d*_*i*_|*t*) is the probability that data source *i *supports *t *when the gene belongs to *t*; it is estimated by the ratio *p*_*a*_/(*p*_*a *_+ *n*_*a*_). *P*(¬*d*_*i*_|¬*t*) is the probability that data source *i *does not support *t *when the gene does not belongs to *t*; it is estimated with *n*_*n*_/(*p*_*n *_+ *n*_*n*_).

Thus, from the three above equations, the conditional probability of *t *can be roughly estimated and it constitutes our global confidence score. This score reflects the likelihood of the predictions: genes with high (near 1) confidence scores are more likely to be associated with *t *than genes with low (near 0) confidence scores. However, due to the independence assumption, this score cannot be interpreted as the probability of *t*. Hence, it is discretized in 4 score categories (very low [0.0, 0.25], low ]0.25, 0.5], high ]0.5, 0.75], and very high ]0.75,1.0]). The true discovery rate associated with each category is estimated by way of a last cross-validation procedure: this is done by computing the proportion of successes among already characterized genes that have been predicted in the considered GO term with a confidence score in this category. These cross-validated true discovery rates then represent our *GDB*. For example, a prediction associated with a *GDB *of 80% means that 80% of the predictions belonging to the same score category in this GO term are correct in the CV procedure. As for the *TDR*s, we also compute the p-value of the *GDB*s. If this p-value is higher than 5%, then the *GDB *is not considered to be significantly higher than the prior probability of the term, and it appears in gray in PlasmoDraft.

The discretization procedure we use, sometimes known as the *equal interval width *method, could be replaced by other methods, such as the *equal frequency interval *method or more sophisticated methods based on entropy minimization [23]. However, it is a classical and simple method that has shown to give good performance on numerous data sets [24].

The independence assumption is often used in statistical machine learning, and forms the basis of the "naive Bayes" predictor, which was shown to be fairly accurate in a number of applications [19]. One interesting feature of this predictor (and hence of the *GDB*) is that it is not much affected by irrelevant or poor quality data sources [25]. Indeed, when a source *i *is not relevant for a specific GO term *t*, either because it has not been designed for screening this type of information or because of the poor quality of the data, terms *P*(*d*_*i*_|*t*) and *P*(*d*_*i*_|¬*t*) tend to be equal. Therefore, the numerical quantities related to this data source tend to cancel in the numerator and denominator pairs of the confidence score. This prevents the *GDB *from pollution by irrelevant or too noisy data sources.

## Results

### Data

To produce the PlasmoDraft database, Gonna has been applied to most publicly available postgenomic data sources we were aware. 9 transcriptomic (microarray), 1 proteomic (mass-spectrometry), and 1 protein-protein interaction data sets were used. Below is a short description of each data set, indexed by the name used in PlasmoDraft.

• LE03: Le Roch et al. (2003) data set [14]. A transcriptomic data set that covers 9 stages of the entire cycle of strain 3D7: 6 asexual intraerythrocytic stages, plus the merozoite, gametocyte, and salivary gland sporozoite stages. Measurements for ~5 100 genes.

• YO3D7: Young et al. (2005) data set [26]. A transcriptomic data set that covers the sexual developmental cycle (gametocytes) of strain 3D7. Measurements for ~5 100 genes.

• YONF54: Young et al. (2005) data set [26]. Same data set as Y03D7, for strain NF54.

• LLHB3: Llinas et al. (2006) data set [27,28]. A transcriptomic data set that covers 48 h of the intraerythrocytic developmental cycle of strain HB3. Measurements for ~4 200 genes.

• LLDd2: Llinas et al. (2006) data set [28]. Same data set as LLHB3, for strain Dd2.

• LL3D7: Llinas et al. (2006) data set [28]. Same data set as LLHB3, for strain 3D7.

• DA06: Dahl et al. (2006) data set [29]. A transcriptomic data set that covers two 48 h life cycles of doxycyclin treated parasites. Measurements for ~5 300 genes.

• SH07: Shock et al. (2007) data set [30]. A transcriptomic data set analysing mRNA decay during the intraerythrocytic developmental cycle. Measurements for ~5 300 genes.

• LE07: A transcriptomic data set analysing the parasite response to choline analog T4 during the intraerythrocytic life cycle. See series GSE4582 in the NCBI Gene Expression Omnibus . Measurements for ~5 100 genes.

• LE04: Le Roch et al. (2004) data set [31,32]. A proteomic data set that covers 7 stages of the entire cycle of strain 3D7: the ring, trophozoite, schizont, merozoite, gametocyte, gamete, and salivary gland sporozoite stages. Measurements for ~2 900 genes.

• LA05: LaCount et al. (2005) data set [33]. A protein-protein interaction data set. Measurements for ~1 300 genes.

The Gene Ontology file (revision 5.754) and the gene annotations file (revision 1.54) were downloaded from the GO website.

### Accessing the database

Users can access the predictions by browsing the database or querying for a specific gene, GO term, or keyword. Results are displayed using three types of views: a global view, a gene view, and a GO term view. In each view, *TDR*s and *GDB*s are represented with a color code that ranges from red (0%) to light green (100%) via yellow (50%); non-significant *TDR*s or *GDB*s (see Method) are in gray.

#### The global views

There is one global view for each gene ontology (Molecular Function, Biological Process, and Cellular Component). A global view (see Figure 2) shows all GO terms of the selected ontology where predictions are made. These are represented in a hierarchical way which respects the ontology structure. Each term is followed by its prior probability, the best *GDB *found for a gene predicted in this term, and the best *TDR *associated with each data source for this term.

#### The GO term view

**Figure 3:**
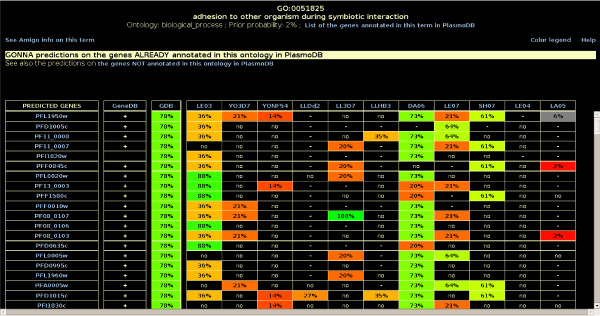
**An extract of the predictions achieved in term "adhesion to other organism during symbiotic interaction" (GO:0051825)**. The "no" entry indicates that the data source does not support the prediction, while "-" means that no data are available in the source for this gene. By clicking on a *TDR*, the *K *characterized nearest neighbors that support/do not support this prediction are shown (see Figure 1). Clicking on any gene opens the corresponding gene view.

The GO term views show all genes that are predicted in any given term by Gonna (see Figure 3). Two views are available for each GO term: one for uncharacterized genes that have no annotation in GeneDB for the ontology at hand, and the other one for genes that are already annotated in this ontology in GeneDB (but not obligatory with this term). For the latter, a '+' symbol after the gene name indicates that the gene is already annotated by the term. Additional information about predictions is provided by clicking on a specific *TDR*. This opens a new window presenting the *K *genes that support, or do not support, the prediction for the corresponding data source, along with their associated profiles (for the transcriptomic and proteomic sources, see Figure 1). A link towards the AmiGO website  allows the user to quickly retrieve additional information on this term.

#### The gene view

**Figure 4:**
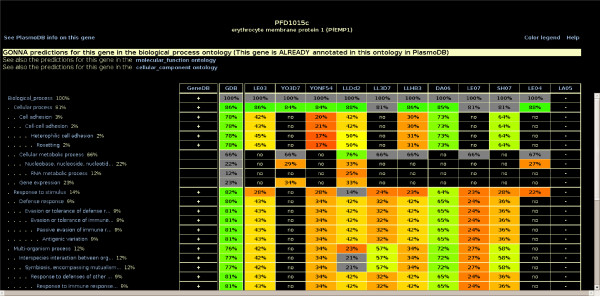
**An extract of predictions achieved for gene PFD1015c in the BP ontology**. The "no" entry indicates that the data source does not support the prediction, while "-" means that no data are available in the source for this gene. By clicking on a *TDR*, the *K *characterized nearest neighbors that support/do not support this prediction are shown (Figure 1). Clicking on any term opens the corresponding GO term view.

The gene view displays the different GO terms that are predicted for each gene by Gonna. These terms are shown in a hierarchical way which follows the ontology structure (see Figure 4). There are three gene views for each gene, which correspond to the three GO ontologies. Each term is followed by its prior probability, the *GDB *of the prediction, and the *TDR*s associated with all data sources that support it. Moreover, for genes that already possess GeneDB annotations in the selected ontology, a ' + ' symbol after the term name indicates that this term already annotates this gene in GeneDB. As for the term view, clicking on a specific *TDR *opens a new window that provides additional information about the corresponding prediction. A link to PlasmoDB allows the user to quickly retrieve additional information on this gene. Note that *TDR*s and *GDB*s associated with the terms usually increase when scrolling toward the top of the ontology, because the prior probabilities of the terms increase. However, they may also decrease sometimes: If a GO term *t *is a generalization of one term *t' *with a good postgenomic signature (high *TDR*) and one term *t" *with a poor signature (low *TDR*), genes predicted in *t" *may have an unfavorable impact on the *TDR *estimation of *t *which may be lower than that of *t'*.

## Discussion

### Annotation quality

Quantity and quality of the available annotations used in the prior knowledge database to generate the predictions is a key point of any GBA approach. For *P. falciparum*, both quantity and quality are questionable. For example, in the BP ontology, of the 1799 genes (35%) possessing annotations, only 228 (13%) have annotations with experimental evidence; annotations of the 1571 remaining genes come from sequence similarity with proteins of other organisms (ISS and IEA evidence codes), and for 1067 genes (68%) these annotations have IEA code, indicating that they have not been reviewed by a curator. Moreover, of the 431 different BP GO terms associated with the *P. falciparum *genes when considering all annotations, 172 (40%) are associated with IEA annotations only. For example, all annotations involving BP GO terms ATP biosynthetic process (GO:0006754), immune response (GO:0006955) or methylation (GO:0032259), as well as their descendants terms, possess IEA code only. Hence, we decided to consider all available GO annotations when generating the PlasmoDraft database. Removing all non-curated annotations from the prior knowledge database would eliminate not only numerous characterized genes, but also numerous GO terms, which would render impossible any new prediction in these parts of the ontology.

### Experiments on a well annotated organism

**Figure 5:**
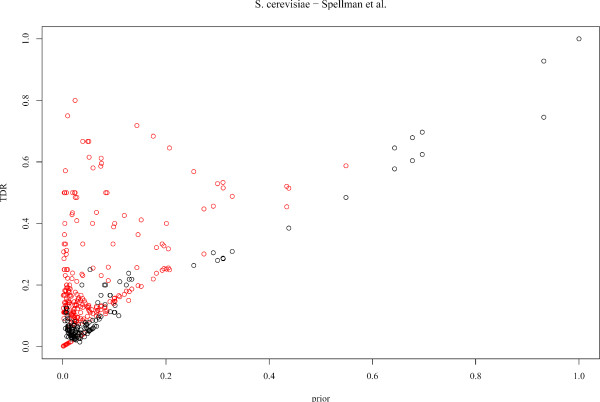
**Gonna performance on yeast**. Gonna was applied to the transcriptomic data set published by Spellman et al. (1998) [34] using experimental evidence code annotations only as prior knowledge database. *TDR*s of all BP GO terms where predictions are proposed by Gonna are plotted as a function of the prior probability of the terms. Red and black points indicate significant and non-significant *TDR*s, respectively.

**Figure 6:**
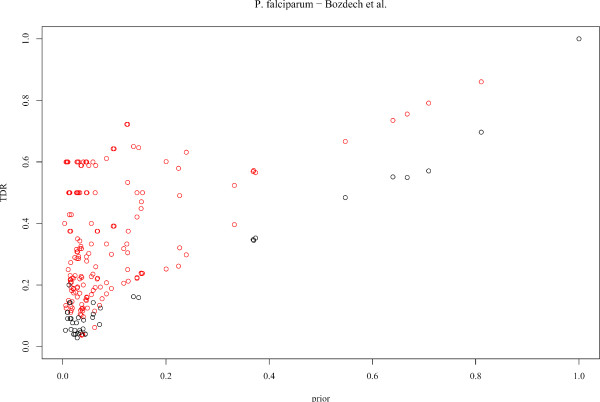
**Gonna performances on the transcriptomic data set published in Bozdech et al. (2003) **[27]. *TDR*s of all BP GO terms where predictions are proposed by Gonna are plotted as a function of the prior-probability of the terms. Red and black points indicate significant and non-significant *TDR*s, respectively.

In these conditions, it was relevant to check the method on a well annotated organism, using only experimental evidence code annotations as input for the predictions and for estimating the *TDR*s. To this end, we applied Gonna on the transcriptomic data set published by Spellman et al. (1998) [34], which monitors the expression level of yeast genes along the cell cycle. The same parameters as for *P. falciparum *were used, *i.e*. neighbor genes were selected using the Pearson correlation coefficient and we used two sets of parameters (*K*, *K'*): (*K *= 6, *K' *= 4) and (*K *= 6, *K' *= 2). All annotations different from IEA, ISS and RCA were used (gene annotation file revision 1.1323, downloaded from the GO website), which involves 4 165 genes characterized in the BP ontology, and a total of 1 220 different GO terms. The *TDR*s were estimated for each GO term by cross-validation. Figure 5 represents the *TDR*s associated with all BP GO terms where predictions are proposed by Gonna, as a function of the prior probability of the terms. We see that for numerous terms, predictions are made with a *TDR *significantly higher than the prior probability of the term, which shows the potential of the approach to decipher biological functions from gene expression data. For comparison purpose, the same experiment was achieved on *P. falciparum *with the time series of Bozdech et al. (2003) [27] using all available BP annotations (see Figure 6). While, as expected, the number of GO terms where predictions are made is lower than for yeast, numerous GO terms are also predicted with high *TDR*s. Though the reliability of these predictions could depends on the prior (IEA) annotations, the similarity of Figures 5 and 6 is quite encouraging and shows that *P. falciparum *annotations are globally consistent, as they are mostly recovered using a transcriptomic data set.

### Contents of the database

By browsing the PlasmoDraft database, several predictions clearly involve rare GO terms (*i.e*. with low prior probability) with high *TDR*s or *GDB*s. For example, in the BP ontology, 16 uncharacterized genes are predicted in establishment of localization (GO:0051234) (prior probability 15%, *GDB *78%), 25 uncharacterized genes are predicted in Rosetting (GO:0020013) (prior probability 2%, *GDB *78%), and 50 uncharacterized genes are in Pathogenesis (GO:0009405) (prior probability 4%, *GDB *75%). Similarly (but with lower *GDB*s), 13 uncharacterized genes are predicted in Ubiquitin-dependent protein catabolic process (GO:0006511) (prior probability 2%, *GDB *50%), and 12 uncharacterized genes are in Biopolymer catabolic process (GO:0043285) (prior probability 3%, *GDB *56%). Moreover the best *TDR*s are not always achieved with the same data source. For example, for the Antigenic variation (GO:0020033) term, the LE03 data [14] provides more accurate predictions than the LLHB3/LLDd2/L13D7 series [27,28], may be because this function has a more apparent expression signature when considering the entire life cycle of the organism. For functions such as DNA packaging (GO:0006323) however, the highest *TDR *is achieved with the LLHB3 data set [27] because the function is better monitored at the cell cycle level.

**Figure 7:**
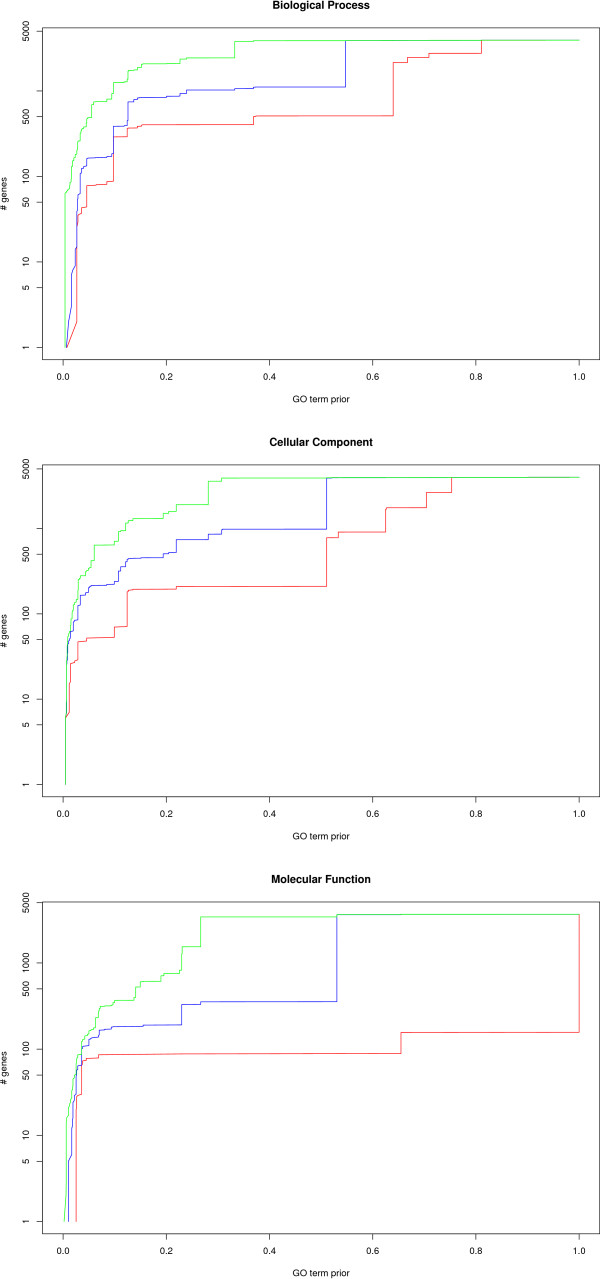
**Estimate of the amount of new information supplied in PlasmoDraft**. Estimates for the BP (up) CC (middle) and MF (down) ontologies. Red, blue and green lines represent the results achieved with *GDB *thresholds of 75%, 50% and 25%, respectively. The *x*-axis gives the prior probabilities of the terms, while the *y*-axis (in log scale) reports the number of uncharacterized genes in the ontology that have been predicted with a *GDB *above the threshold, on a GO term with prior probability below *x*.

We estimated the amount of new information provided by PlasmoDraft in a systematic way. For the BP ontology, PlasmoDraft proposes significant annotations on GO terms of low prior probability (below 25%) for 3 900 genes, among which 2 434 have no BP annotations in GeneDB. With CC and MF ontologies, 1 905 and 1 540 uncharacterized genes are annotated by PlasmoDraft on low prior probability GO terms, respectively. The interest of these annotations of course depends on the associated *GDB*. Thus, given a *GDB *threshold (*e.g*. 75%) and an ontology, for each uncharacterized gene in this ontology we searched the GO term with the lowest prior probability wherein the gene is predicted with a statistically significant *GDB *above the threshold. Figure 7 summarizes these results on the three ontologies. From this figure we see, for example, that for the BP ontology 290 uncharacterized genes in GeneDB are predicted with a *GDB *above 75% (red curve) on a GO term with a prior probability below 0.10. In the same manner, 1 025 uncharacterized genes are predicted with a *GDB *above 50% (blue curve) on a GO term with a prior probability below 0.25. For the CC and MF ontologies, 740 and 329 genes are predicted with a *GDB *above 50% on a GO term with a prior probability below 0.25, respectively. Note that only genes without any annotation in GeneDB in the selected ontology are considered in this measure, while the PlasmoDraft database also provides additional annotations for many genes that are already annotated in this ontology. By comparing the results achieved on the different ontologies, we see that the BP ontology provides the best results. This is not surprising, as the signature detected in the postgenomic data by GBA methods are mostly characteristic of biological processes [8]. However, by an information propagation phenomenon, the BP signatures may sometimes help for predicting annotations in the two other ontologies. This happens, for example, when many genes with a given molecular function (or exported in a particular cellular component) are involved in a biological process with a strong signature. For example. GO term host cell plasma membrane (GO:0020002) in the CC ontology is associated with high *GDB *(72%), because most genes belonging to this term are also associated with the biological process Defense response (GO:0006952) which is well recognized.

**Figure 8:**
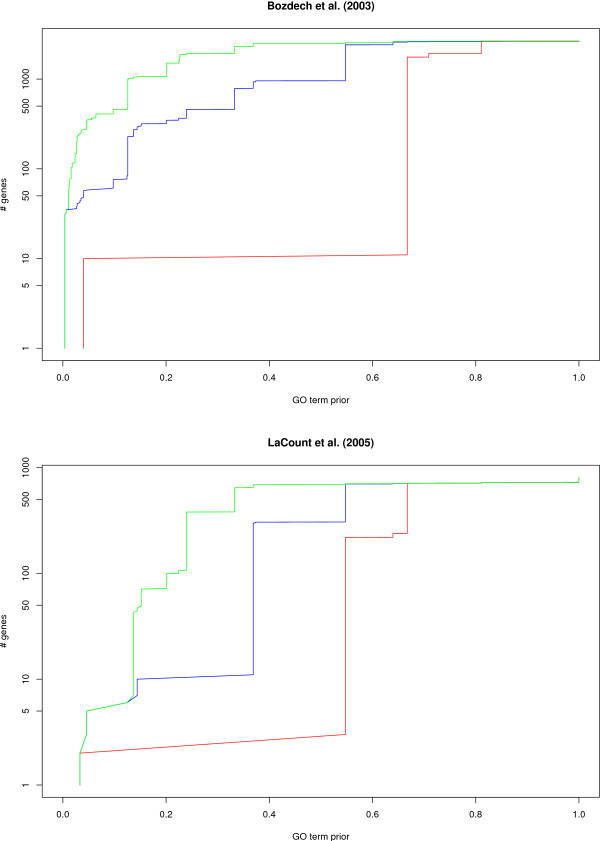
**Estimate of the amount of new information supplied by the transcriptomic data source of Bozdech et al. (2003) **[27]** and the interactomic data source of LaCount et al. (2005) **[33]. Red, blue and green lines represent the results achieved with *TDR *thresholds of 75%, 50% and 25%, respectively. The *x*-axis gives the prior probabilities of the terms, while the *y*-axis (in log scale) reports the number of uncharacterized genes in the ontology that have been predicted with a *TDR *above the threshold, on a GO term with prior probability below *x*.

A similar approach can be used to estimate the amount of new information provided by each data source independently. For example, Figure 8 reports the number of uncharacterized genes in the BP ontology that can be annotated with a *TDR *above 75%, 50% and 25% by the transcriptomic data of Bozdech et al. (2003) [27], and by the interactomic data of LaCount et al. (2005) [33]. We can see that more than 73 genes are associated with a GO term of prior probability below 10% with a TDR above 50% using the transcriptomic data, while 10 genes only are predicted with the same thresholds using interactomic data. This indicates that the interactome tends to provide less functional signal than the transcriptome, partly because less genes are monitored.

### Assessment of the *GDB*s

*TDR*s and *GDB*s are estimated by cross-validation by applying Gonna on the already characterized genes. This procedure produces unbiased estimates of the method accuracy, provided that the uncharacterized genes share approximately the same distribution as the characterized ones [19]. However, since *TDR*s and *GDB*s are sometimes estimated on small numbers of predictions, users should be aware that for some specific GO terms, the accuracy on the uncharacterized genes may differ from the reported *TDR*s and *GDB*s. Nonetheless, these measures provide valuable indications on the potential functions of genes by pointing out the most likely GO terms. To assess this point, we compared the PlasmoDraft predictions proposed for the uncharacterized genes to the annotations of their homologous genes in yeast when these are known. We looked on the 986 genes without BP annotations that have been predicted with high *GDB *(above 50%) on specific BP terms (prior probability below 25%). As expected, few genes among these 986 can be associated with a characterized orthologous gene in *S. cerevisiae*. However, a reciprocal best hit procedure using BLASTP with an e-value cutoff of 10^-5 ^allows to find *S. cerevisiae *orthologues for 141 genes. Among these 141 orthologous pairs, 63 (45%) have "concordant" annotations with the high *GDB *predictions. Here we consider that annotations are concordant if at least half of the terms with prior probability below 25% are shared by the *S. cerevisiae *orthologue. As expected, this proportion decreases when using PlasmoDraft predictions with lower *GDB*s. For example, 2 271 genes without BP annotations are predicted with a *GDB *between 25% and 50% on a GO term with prior probability below 25%. Among these, 245 can be associated with *S. cerevisiae *orthologues by reciprocal best hit, and 71 (29%) have concordant annotations.

### Comparison with the predictions of Zhou et al. (2008) [35]

During the writing of this article, another database [35] of gene function predictions based on the OPI method described in reference [17] was published. Briefly, OPI is a supervised method that works as follows. For each GO term, OPI uses a set of "seed" genes already annotated with this term to construct an average expression profile. Next, all genes (annotated or not) are ranked according to their similarity to this average profile and a statistical test is used to identify the rank cutoff that includes the largest number of seed genes within the smallest cluster size. All genes before this cutoff are then considered as potentially related to the GO term under consideration. The database [35] exploits a single new transcriptomic data set covering all life cycle stages of the parasite and combining gene expressions from both *P. yoelii *and *P. falciparum*. As both the methods and data sources are different, this database and PlasmoDraft provide different and complementary information. OPI provides BP annotations for 1 902 different genes, among which 1 036 have no BP annotations in GeneDB. When looking at the PlasmoDraft predictions with *GDB *above 50% (which involves 1 111 uncharacterized genes in BP), only 230 also have BP predictions in OPI. However, when both methods propose BP predictions for a gene, the predictions are often similar. Indeed, of the 230 common genes, 94 have concordant predictions – *i.e*. at least half of the predictions of one of the methods involving terms with a prior probability below 25% are also predicted by the other method. Differences in specificity of OPI and PlasmoDraft can also be observed by comparing the *GDB *and *FDR *(false discovery rate) estimates associated with a given GO term by PlasmoDraft and OPI, respectively. Recall that the *GDB *is actually the *TDR *associated with the predictor that combines all data sources. Moreover, by definition, the *FDR *equals 1 minus the *TDR *on this term. While *FDR*s of OPI are not estimated by cross-validation, we can nevertheless get a rough idea of which method provides the best results for a given GO term. For example, OPI obtains the highest *TDR*s on terms like Entry into host (GO:0044409), or Mitochodrion organization and biogenesis (GO:0007005) (~90% and ~30% vs. 36% and ~5%), while for terms like Interaction between organisms (GO:0044419) or Rosetting (GO:0020013), PlasmoDraft obtains the best results (77% and 78% vs. 25% and no statistically significant *TDR*). On the whole, it thus appears that the two databases use quite different data sources and provide interesting information on different types of functions and different genes, so the community will likely benefit from both.

## Conclusion

We presented PlasmoDraft, an extensive database of GO annotation predictions that are achieved by Guilt By Association using most postgenomic data available to date for *P. falciparum*. All predictions come with a confidence estimate computed by cross-validation. The database is presented in a friendly interface that allows easy browsing and querying, and proposes high confidence annotations for several hundreds of genes without any annotations, as well as additional annotations for many already characterized genes. One prospect is the integration of *compendiums *of gene expression data sets as new data sources in PlasmoDraft. These data, obtained by concatenation of several data sets of diversified biological conditions, have shown to often provide strong biological function signatures [36]. However, predictions based on these data may be difficult to interpret for biologists, and their integration opens new issues in data selection and combination.

As mentioned in the Methods, one advantage of Gonna concerns its genericness that allows its use on any new data, as long as a relevant similarity measure can be computed; a set of scripts then enables regeneration of the database to integrate the new data set in a fully automated way. This also holds for the GO annotations used as prior knowledge, and the new annotations provided by the community in the future will be easily integrated. Most notably, we are aware that a collegiate effort for re-annotating *P. falciparum *proteins should provide new/curated functional annotations in the near future. This should improve both the quantity and the quality of the PlasmoDraft predictions. In the same way, while in the current version of PlasmoDraft all GO annotations are considered (*i.e*. including automatically-assigned annotations) due to the scarcity of curated annotations, it is possible that the re-annotation effort will enable the use of only curator-assigned annotations in the subsequent versions of PlasmoDraft. Thanks to these new advances, PlasmoDraft should become more and more accurate and useful to the community.

## Availability and requirements

PlasmoDraft is freely available at 

## Authors' contributions

LB conceived, designed and implemented the method, carried out the analyses, designed the database and drafted the manuscript. JFD designed and developed the database. OG initiated the project, designed the method, participated in the analyses, designed the database and revised the manuscript. All authors read and approved the final manuscript.
